# A bibliometric analysis of the 100 most cited articles describing SARS-CoV-2 variants

**DOI:** 10.3389/fpubh.2022.966847

**Published:** 2022-08-26

**Authors:** Yahui Zhang, Meijing Feng, Yongmei He, Fangming Liu, Rui Ma

**Affiliations:** Department of Health Management, Aerospace Center Hospital, Beijing, China

**Keywords:** SARS-CoV-2, infection, variant, mutation, bibliometric analysis, public health

## Abstract

**Background:**

The emergence of severe acute respiratory syndrome coronavirus 2 (SARS-CoV-2) variants with mutations in the spike protein has risen concerns about the efficacy of infection- or vaccine-induced antibodies and has posed a serious threat to global public health, education, travel and economy. Few studies have described the detailed characterizations of highly cited articles on SARS-CoV-2 variants.

**Objective:**

To identify and characterize the 100 most-cited articles in SARS-CoV-2 variants research.

**Design and methods:**

Articles published recently were extracted from the web of science core collection database using a query based on MeSH terms and topics of SARS-CoV-2 and variants. Characteristics of the 100 most-cited articles were analyzed *via* the following parameters: publication number over year, number of citations, type of articles, authors, journal, journal impact factor, country, and topics covered in articles. In addition, clinical trials in these articles were also analyzed.

**Results:**

The majority of articles (66%) were published in 2021. Number of citations of the 100 most cited articles ranged from 1720 to 75 (median: 178.5). Mutations in the S protein such as D614G mutation and the B.1.1.7 (UK) and B.1.351 (South Africa) were the dominant variants in the 100 most cited articles. The United States, the United Kingdom, and South Africa had the strongest collaboration in the contribution of publication. Science, Cell, Nature and New England Journal of Medicine were mostly cited and the main direction in these top journals were vaccine neutralizing tests and efficacy evaluation studies. Response of antibody neutralization tests against variants was always weakened due to the presence of variants but the results of clinical trials were encouraging. Genomics information, spike protein structure confirmation and neutralization studies evaluating antibody resistance were highly represented in the 100 most cited articles in SARS-CoV-2 variants literature.

**Conclusions and relevance:**

Altogether, genomic information, epidemiology, immune neutralization, and vaccine efficacy studies of COVID-19 variants are the main research orientations in these articles and relevant results have been published in influential journals. Given the continuous evolution of the SARS-CoV-2 and the constant development in our understanding of the impact of variants, current working strategies and measures may be periodically adjusted.

## Introduction

Severe acute respiratory syndrome coronavirus 2 (SARS-CoV-2), the virus that causes COVID-19, experiences changes in gene structure at any time (1). So far, this pandemic has severely threatened global public health. The best way to prevent and slow down transmission is to be well-informed about the disease and how the virus spreads. However, the emergence of the new variants brings big challenges to the therapeutic and preventive strategies of COVID-19 (2–5). The World Health Organization recently reported that there were 14.9 million excess deaths due to SARS-CoV-2 pandemic in 2020 and 2021 (https://www.who.int/zh). In the past few months, researchers identified that these emerging variants were caused by genome mutations and were emerging rapidly, as exemplified by the B.1.1.7 (United Kingdom), B.1.351 (South Africa), and P.1 (Brazil) lineages termed variants of concern/interest (VOC/I) because of the greater risk they pose due to possible enhanced transmissibility and infectivity, immune response evasion, treatment failure, and reduced vaccine efficacy (6–8). Rapid development and evaluation of medical countermeasures against new emerging SARS-CoV-2 variants are urgently needed.

Ever since the initial discovery and identification, more than three thousand studies on this domain have already appeared in the scientific literature. Published articles covered a wide range of topics, including but not limited to, cell biology, biochemistry molecular biology, medicine general internal, microbiology, virology, and infectious diseases. Concurrently, these articles also revealed a wide range of dominant countries, journals, institutions, and authors that have made certain contributions in this field. Given the broad range of interests covered in these published works, it is of great necessity for scientific investigators to determine the precise study direction and relative challenges. Characterization of high-quality studies can aid researchers to tailor their study orientations and pursuit to associated interests.

Bibliometric science is mainly composed of citation analysis which is the source of impact factors (9). Publications with high citation counts are identified as core to a specific field. Therefore, highly cited articles can provide key information about research trends and scientific advances in a given field (10). To our best knowledge, few studies have reported important characterizations about the top 100 cited studies on SARS-CoV-2 variants. Our study did not seek to determine the types of studies being published; rather, we sought to determine the characteristics of highly cited articles.

Here, we aimed to identify the 100 most cited articles focused on SARS-CoV-2 variants and to characterize these articles.

## Methods

### Articles search and selection

**Figure 1:**
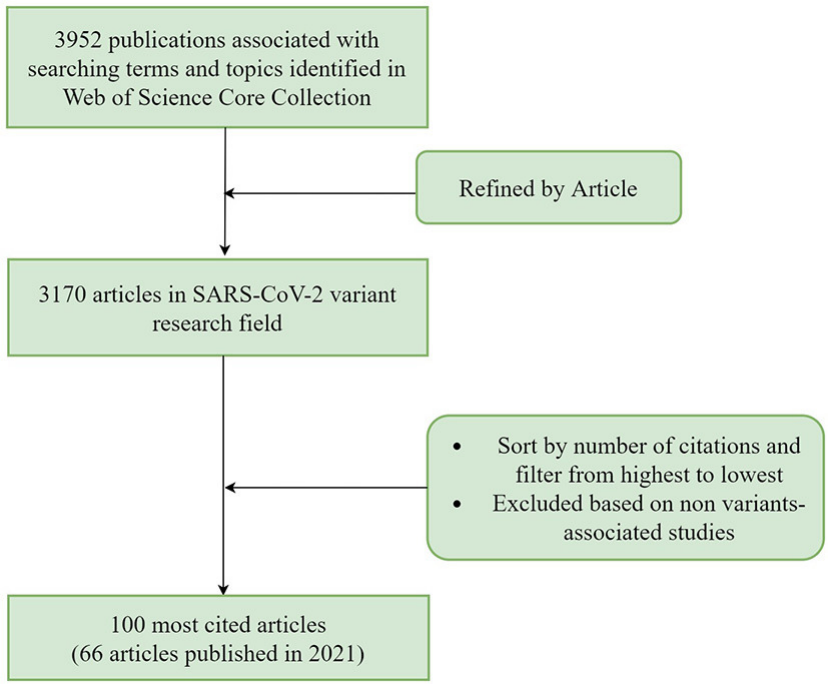
Study flow chart.

On May 24, 2022, we generated a series of studies related to SARS-CoV-2 variants by utilizing the Web of Science Core Collection database. We used the following search terms and topics extracted from Medical Subject Headings (MeSH) to retrieve publications in the database: (TS = (SARS Coronavirus 2 OR 2019 Novel Coronavirus OR SARS-CoV-2 Virus OR 2019-nCoV OR Wuhan Coronavirus OR COVID19 Virus)) AND TS = (variant OR variants). Next, we selected “Articles” for the file type and the publication period from January 1, 2020 to May 24, 2022 for the search and obtained 3,170 original articles. Next, we sorted these articles in descending order of citations and checked whether they were related to the SARS-CoV-2 variant. Through this flow (Figure 1), we finalized the top 100 most cited articles on SARS-CoV-2 variants.

Two reviewers (Zhang and He) performed independent article identification and analysis, and a third senior reviewer (Ma) reassessed the results and discussed with them until a consensus was reached.

A SARS-CoV-2 variant article was defined as any research primarily focusing on emerging variants genomic information and epidemiology, special structure confirmation, vaccine design and development, randomized controlled trials on safety and efficacy of vaccine and animal studies about neutralizing antibody tests. Articles unrelated to the SARS-CoV-2 variant were excluded.

### Data extraction and analysis

All of the 100 most cited articles were available online in the MEDLINE database. Every article was reviewed for retrieval of the publication year, journal title, journal impact factor, first author, number of citations (total citations), citations over year, type of article (basic research, randomized controlled trial or cross-sectional study), country and main topics covered in the articles. VOS viewer (Leiden University, Leiden, The Netherlands, version 1.6.17), a free JAVA-based software for bibliometric mapping developed by Ludo Waltman, and CiteSpace (version 5.8.R3), a visibility tool created by Prof. Chaomei Chen, were used for data visualization of co-authorship, co-citation, and co-occurrence analysis. Co-cited references (journals, authors) are two (or more) references (journals, authors) cited by one or more articles simultaneously (11, 12). Clustering analysis of the co-citation results can indicate the research categories and directions. In addition, we used co-occurrence analysis (also known as keyword analysis) to cluster keywords from different studies to represent and predict research hotspots in the field (11).

### Statistical analysis

We used SPSS (version 26.0, IBM, Armonk, NY, USA) to describe the data distribution and analyze the correlation between JIF (journal impact factor) and CIF (number of citations/impact factor) ratio. Kolmogorov-Smirnov test was used to test the normality of continuous data. Continuous variables tested for normality were presented as the means and SD, otherwise as the median (interquartile range, IQR). *P* < 0.01 was set as statistical significance.

## Results

**Figure 2:**
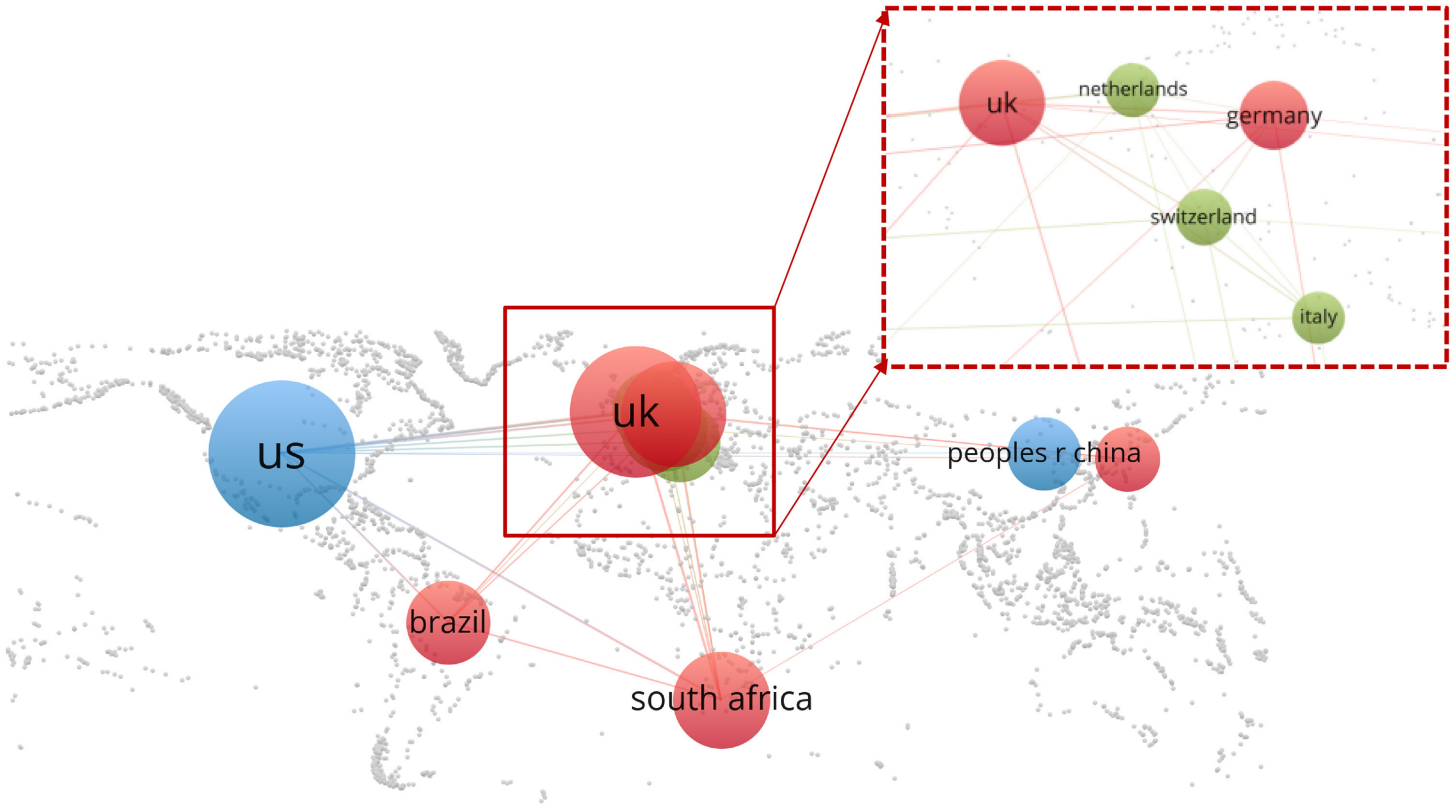
Country collaboration network on SARS-CoV-2 variants. The gray section indicates the national geographic border information.

**Figure 3:**
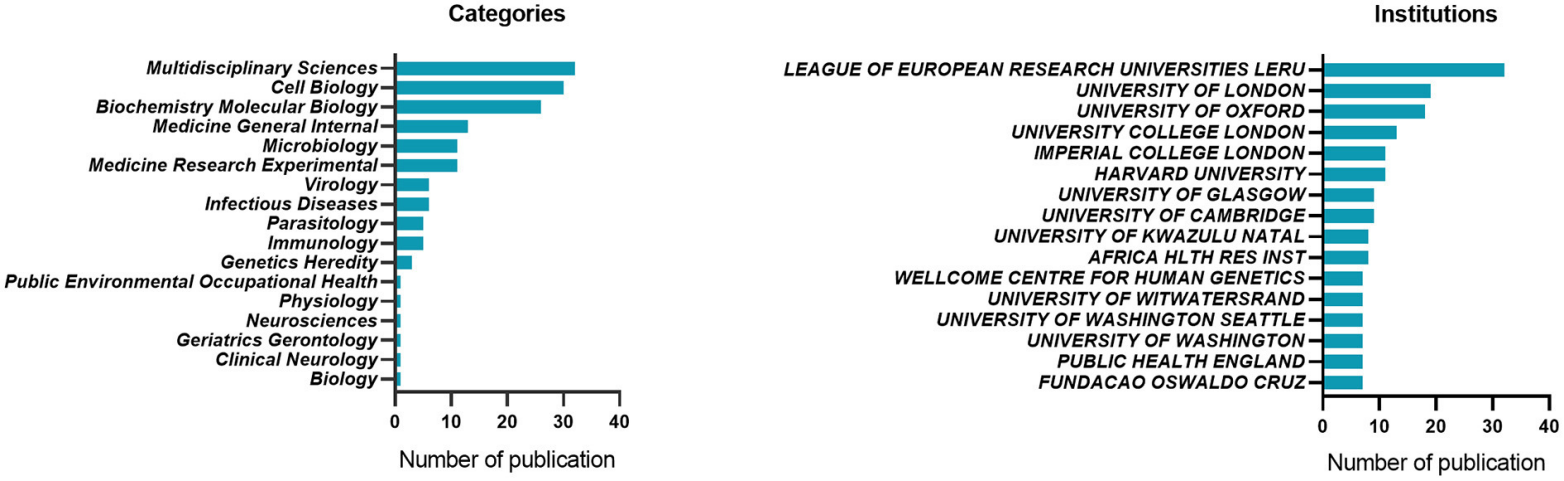
Top 15 institutions and study categories of the total number of publications.

Sixty-six of the 100 most cited articles were published in 2021. A total of 36 countries participated in the publication of the 100 most cited articles. The majority of the publications were contributed by the United States (*n* = 57), distantly followed by United Kingdom (*n* = 36), Germany (*n* = 19) and South Africa (*n* = 14) (Table 1). Coauthor ship analysis of countries showed the strongest collaboration between the United States, the United Kingdom, and South Africa (Figure 2). The highly cited articles were published in 33 journals, led by Nature (*n* = 14) and followed by Cell (*n* = 13), Science (*n* = 13), Nature Medicine (*n* = 10) and New England Journal of Medicine (*n* = 10) (Table 2). The top 15 institutions and study categories of total number of publications were presented in Figure 3.

**Table 1:** Top 10 countries of origin of the 100 most-cited SARS-CoV-2 variant articles.

**Country**	**No. of**	**Total of**	**Average**
	**articles**	**citations**	**citations**
The United States	57	16,201	284
England	36	11,191	311
Germany	19	5,318	280
South Africa	14	4,000	286
Scotland	11	2,837	258
Switzerland	10	2,626	263
Brazil	8	2,436	305
Italy	7	2,083	298
Japan	6	988	165
Netherlands	6	2,282	380
China	6	1,496	249

**Table 2:** Journals in which two or more of the 100 most-cited SARS-CoV-2 variant articles were published.

**Journal**	**No. of articles**	**Total citations**	**Average citations**	**Impact factor (JCR 2020)**
Nature	14	4,272	305	49.962
Cell	13	4,944	380	41.584
Science	13	3,921	302	47.728
Nature medicine	10	2,682	268	53.44
New England journal of medicine	10	3,294	329	91.253
Cell host microbe	5	628	126	21.023
Cell reports	4	465	116	9.423
PNASUSA	3	669	223	11.205
Lancet	2	415	208	79.323
Lancet infectious diseases	2	223	112	25.071
Nature communications	2	359	180	14.919

JCR, Journal Citation Reports; PNASUSA, Proceedings of the National Academy of Sciences of the United States of America.

The number of citations of the 100 most cited articles ranged from 1,720 to 75 (median: 178.5). All of the articles were published in 33 different journals and contributed by 2,129 authors, with an overall 26,153 total citations (10,221 citations in 2020, 15,757 citations in 2021, 175 citations in 2022). Characterization of the top 20 highly cited articles in SARS-CoV-2 variants and their number of citations over years were presented in Table 3 (complete features of 100 top-cited articles were shown in Supplementary Table 1). The top-ranking article of total citations conducted in 2020 by Korber et al. (13) dynamically tracked structural and biological changes in SARS-CoV-2 spike of variants based on regular updates from the GISAID SARS-CoV-2 sequence database and revealed that the spike D614G amino acid change (mutations) was associated with potentially higher viral loads in COVID-19 patients and can increase the frequency and global distribution making the variant carrying the D614G spike mutation became the globally dominant form of SARS-CoV-2.

**Table 3:** The 20 most-cited SARS-CoV-2 variant articles ranked in order of the number of citations received.

**Rank**	**Article**	**Type of article**	**No. of citations**	**Citations over year**			**Author (first)**	**Journal**
				**2020**	**2021**	**2022**		
1	Tracking changes in SARS-CoV-2 spike: evidence that D614G increases infectivity of the COVID-19 Virus	Basic research	1,720	239	1,199	282	Korber, B	Cell
2	Antibody resistance of SARS-CoV-2 variants B.1.351 and B.1.1.7	Basic research	894	0	630	264	Wang, PF	Nature
3	Effectiveness of COVID-19 vaccines against the B.1.617.2 (Delta) variant	Case-control design	870	0	429	442	Bernal, JL	NEJM
4	Inborn errors of type I IFN immunity in patients with life-threatening COVID-19	Basic research	786	58	596	132	Zhang, Q	Science
5	Estimated transmissibility and impact of SARS-CoV-2 lineage B.1.1.7 in England	Basic research	781	1	534	246	Davies, NG	Science
6	Neutralizing antibody levels are highly predictive of immune protection from symptomatic SARS-CoV-2 infection	Basic research	739	0	361	378	Khoury, DS	Nature medicine
7	A multibasic cleavage site in the spike protein of SARS-CoV-2 is essential for infection of human lung cells	Basic research	720	182	416	122	Hoffmann, M	Molecular cell
8	The impact of mutations in SARS-CoV-2 spike on viral infectivity and antigenicity	Basic research	638	44	467	128	Li, QQ	Cell
9	Detection of a SARS-CoV-2 variant of concern in South Africa	Basic research	624	0	446	178	Tegally, H	Nature
10	Safety and efficacy of single-dose Ad26.COV2.S vaccine against COVID-19	Clinical trial	603	2	326	275	Sadoff, J	NEJM
11	Spike mutation D614G alters SARS-CoV-2 fitness	Basic research	601	13	460	128	Plante, JA	Nature
12	COVID-19 vaccine BNT162b1 elicits human antibody and T(H)1 T cell responses	Basic research	556	16	433	107	Sahin, U	Nature
13	SARS-CoV-2 501Y.V2 escapes neutralization by South African COVID-19 donor plasma	Basic research	542	0	423	119	Wibmer, CK	Nature medicine
14	Antibody cocktail to SARS-CoV-2 spike protein prevents rapid mutational escape seen with individual antibodies	Basic research	526	63	375	88	Baum, A	Science
15	Phylogenetic network analysis of SARS-CoV-2 genomes	Basic research	484	221	231	32	Forster, P	PNASUSA
16	Efficacy of the ChAdOx1 nCoV-19 COVID-19 vaccine against the B.1.351 variant	Clinical trial	480	1	345	134	Madhi, SA	NEJM
17	Emerging SARS-CoV-2 mutation hot spots include a novel RNA-dependent-RNA polymerase variant	Basic research	433	132	253	48	Pachetti, M	Journal of translational medicine
18	Genomics and epidemiology of the P.1 SARS-CoV-2 lineage in Manaus, Brazil	Basic research	410	1	284	125	Faria, NR	Science
19	Evidence of escape of SARS-CoV-2 variant B.1.351 from natural and vaccine-induced sera	Basic research	381	0	249	132	Zhou, D	Cell
20	Escape from neutralizing antibodies by SARS-CoV-2 spike protein variants	Basic research	350	2	240	108	Weisblum, Y	Elife

NEJM, New England Journal of Medicine; PNASUSA, Proceedings of the National Academy of Sciences of the United States of America.

Medians (interquartile range, IQR) of the JIF and CIF ratio were 47.728 (35.043) and 5.796 (9.382), respectively. Bivariate correlation analysis (Spearman) demonstrated a significant negative correlation between JIF and CIF ratio (correlation coefficient = −0.677, *p* = 0.000). We further present the top 20 articles in order of CIF from highest to lowest in Supplementary Table 2. The sources of articles and journal impact factors were also summarized in the table.

**Figure 4:**
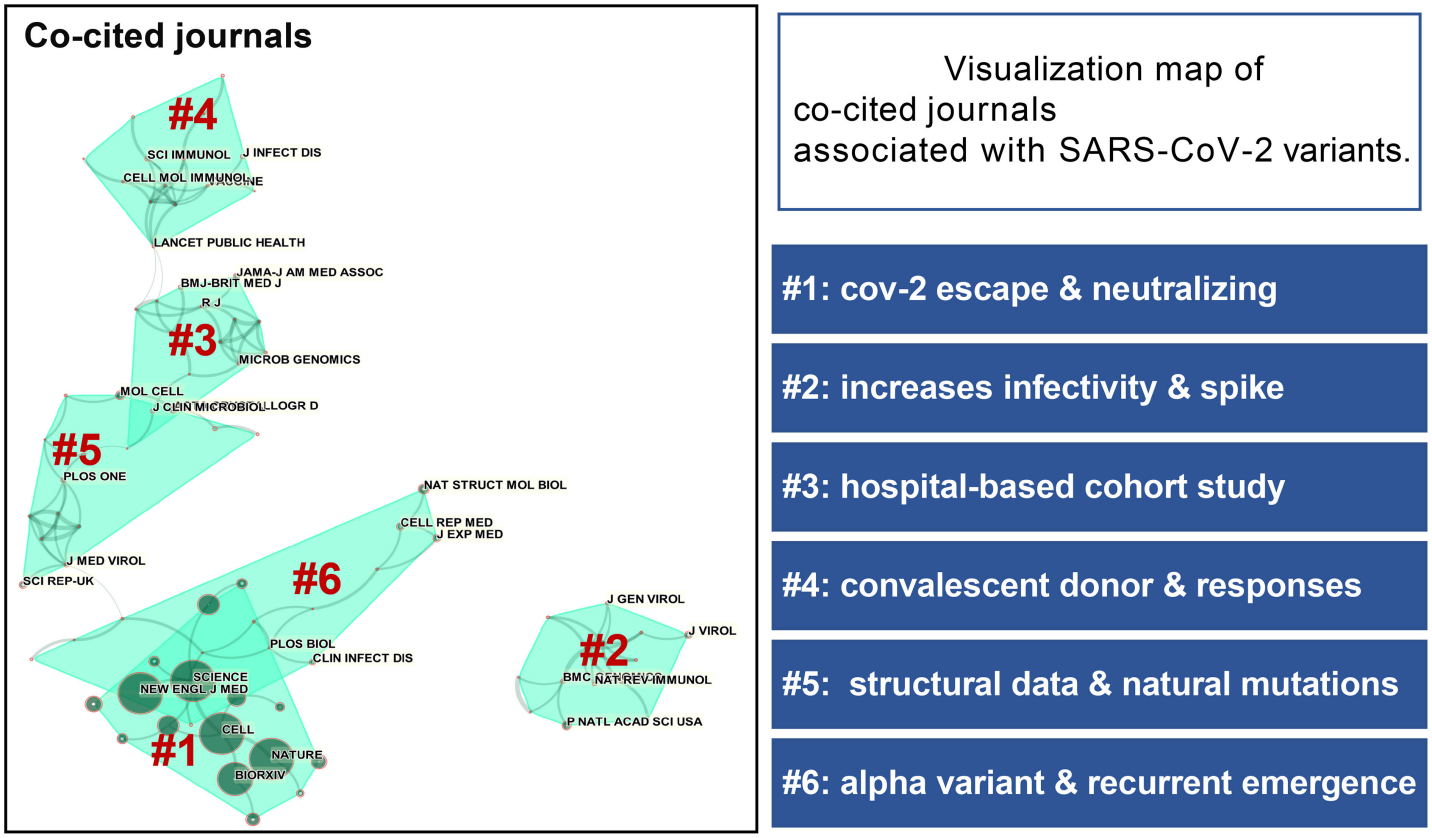
Visualization map of co-cited journals associated with SARS-CoV-2 variants and corresponding clusters based on research direction. The size of the circles indicates the number of co-citations. Lines between categories indicate cross-linkages between study directions.

**Figure 5:**
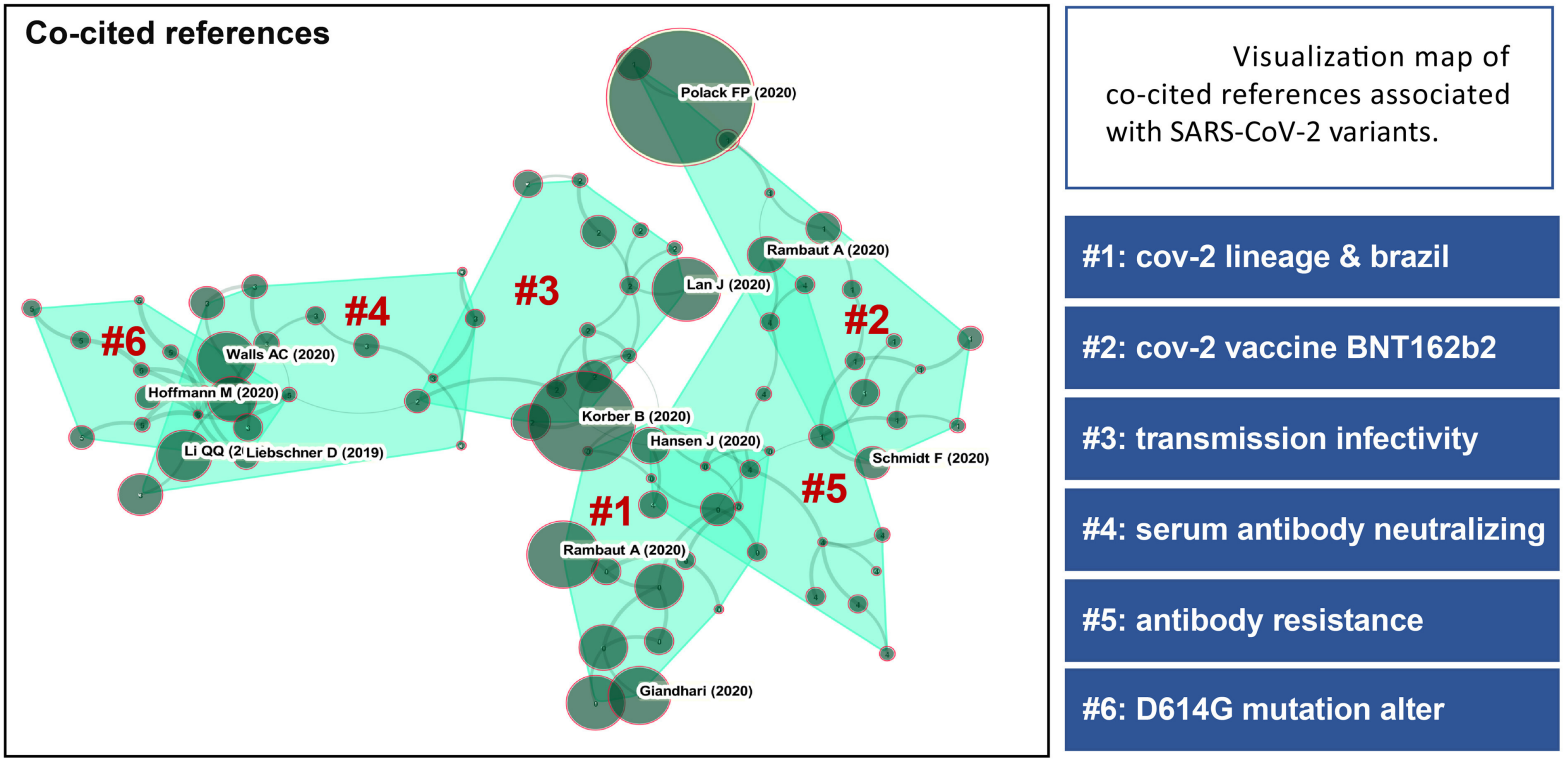
Visualization map of co-cited references associated with SARS-CoV-2 variants and corresponding clusters based on research direction. The size of the circles indicates the number of co-citations. Lines between categories indicate cross-linkages between study directions.

**Figure 6:**
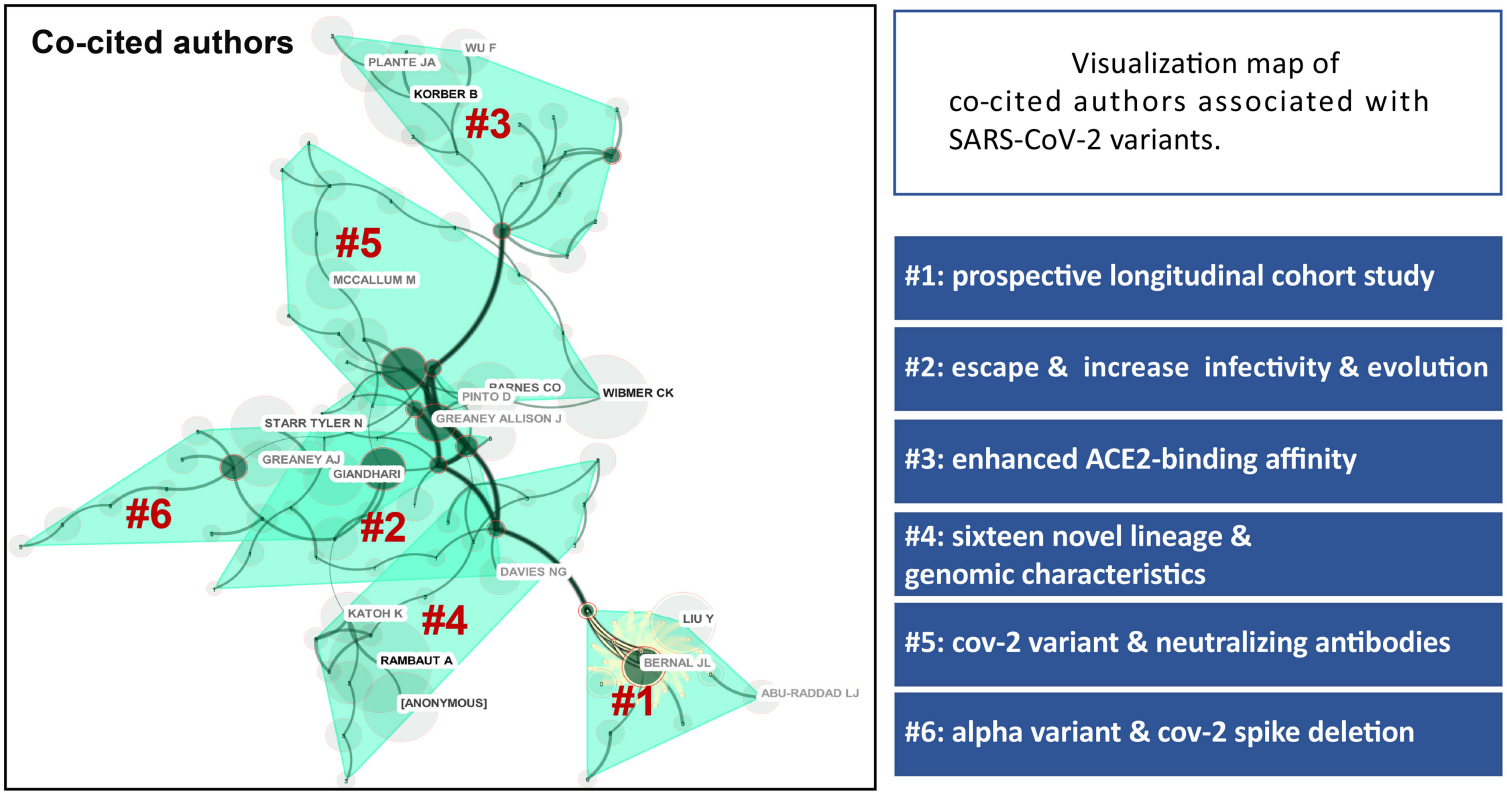
Visualization map of co-cited authors associated with SARS-CoV-2 variants and corresponding clusters based on research direction. The size of the circles indicates the number of co-citations. Lines between categories indicate cross-linkages between study directions.

The most frequently co-cited journal mainly included Science, Cell, Nature and New England Journal of Medicine and most attention in these top journals has been paid to the topics of SARS-CoV-2 escape and neutralizing (Figure 4). The top 5 most frequently co-cited references involved Polack et al. (14), Korber et al. (13), Hoffmann et al. (15), Baum et al. (16), and Rambaut et al. (17). Clustering analysis based on research directions and detailed characterizations of co-cited references were summarized in Figure 5. Accordingly, Polack and Hoffmann had the most co-citations in this domain and the correlation between clustering cores of the most frequently co-cited authors was presented in Figure 6.

**Figure 7:**
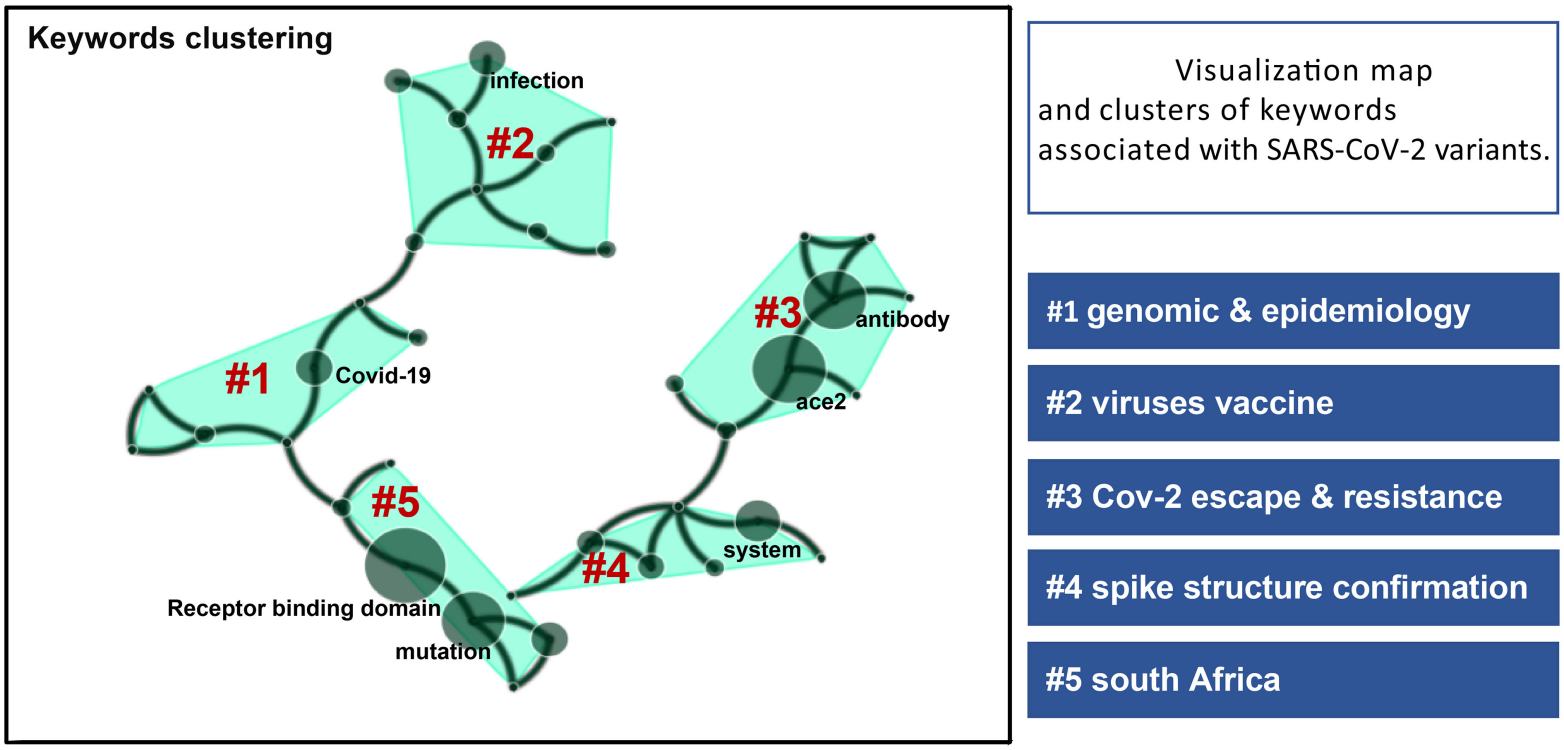
Visualization map and clusters of keywords associated with SARS-CoV-2 variants and corresponding clusters based on research direction. The size of the circles indicates the frequency of keyword occurrences.

Two hundred and thirty keywords were extracted from the 100 cited articles and were classified into five clusters: variants genomic analysis and epidemiology, COVID-19 vaccine effort, immune escape, spike structure confirmation, and South Africa variants studies (Figure 7). Furthermore, a review of articles showed that the most common topics were genomics information and transmissibility (*n* = 30), followed by neutralization studies (*n* = 27), antibody resistance (*n* = 23), and spike protein structure exploration (*n* = 18) (Table 4). Mutations in the S protein such as D614G mutation and the B.1.1.7 (alpha, UK) and B.1.351 (South Africa) were the dominant variants in these articles (Table 5).

**Table 4:** Main topics covered in the 100 most-cited SARS-CoV-2 variants articles.

**Topic and keyword cluster**	**No. of articles**			
	**2020**	**2021**	**2022**	**Total**
	**32**	**66**	**2**	**100**
**#1 Genomic; epidemiology; transmission**	18	11	1	30
Genomics information	13	1	0	14
Epidemiology	1	3	0	4
Transmissibility, antigenicity and global impact	4	7	1	12
**#2 COVID-19 vaccine effort; vaccine effectiveness**	3	24	0	27
Neutralization studies	2	11	0	13
Safety and efficacy evaluation (RCT)	0	6	0	6
booster vaccination and immune memory	1	4	0	5
Vaccine breakthrough infections with variants	0	3	0	3
**#3 Immune escape; resistance**	2	20	1	23
Immune escape (waning)	1	10	1	12
Antibody resistance	1	10	0	11
**#4 Spike structure confirmation; natural mutation**	9	9	0	18
Spike protein structure	6	3	0	9
Evidence that variants increase infectivity (transmissibility)	1	1	0	2
S protein receptor-binding domain mutation	2	5	0	7
**#5 South Africa; Cov-2 variant**	0	2	0	2

**Table 5:** Variant types or associated mutations and their occurrence frequencies in different topic clusters.

**Cluster**	**Topic**	**Variant types or associated mutations**	**Counts**
**# 1**	Genomic; epidemiology; transmission	D614G	6
		B.1.1.7 (alpha, UK)	2
		B.1.617.2 (delta, India)	1
		ACE-2	1
		P.1 variant (Brazil)	1
		ORF1ab 4715L	1
		BA.4 open reading frame 7b (ORF7b) and ORF8	1
**# 2**	COVID-19 vaccine effort; vaccine effectiveness	B.1.1.7 (501Y.V1, alpha, UK)	11
		B.1.351(501Y.V2, SA)	9
		B.1.617.2 (delta, India)	3
		D614G	2
		Wuhan-Hu-1	1
		P.1 variant (Brazil)	1
		B.1.617.1 (India)	1
		B.1.618 (India)	1
		B.1.525 (Nigeria)	1
**# 3**	Immune escape; resistance 2	B.1.351(501Y.V2, SA)	10
		B.1.1.7 (alpha, UK)	4
		P.1 variant (Brazil)	3
		B.1.617.2 (delta, India)	3
		Pango lineage B.1.1.529 (Omicron)	1
**# 4**	Spike structure confirmation; natural mutation	D614G	7
		L452R, T478K, E484Q, P681R, and so on (spike mutations)	2
		ACE-2	2
		B.1.1.7 (alpha, UK)	2
		B.1.351 (SA)	1
**# 5**	South Africa; Cov-2 variant	B.1.351 (SA)	2

SA, South African.

Six clinical trials were conducted to explore the efficacy and safety of existing vaccines against the dominant variants such as B.1.1.7 (United Kingdom), B.1.351 (South Africa) lineages. Variants lineages, number of subjects, vaccine types, main clinical measure and National Clinical Trial (NCT) number were summarized in Table 6. It is noteworthy that all randomized controlled trials were reported in 2021 and 5 of them were phase 3 trials. Median (range) of the number of patients with evidence of SARS-CoV-2 variants infection and durations in these trials were 35 (8–520) persons and 28 (7–180) days, respectively. It is noteworthy that the primary clinical measures of 4 of 6 RCTs were mainly concentrated on the efficacy evaluation of a two-dose regimen (or booster injection) of vaccines against B.1.1.7 variants or B.1.351 variants. Most of their results were encouraging in addition to the clinical trial conducted by Madhi et al. (19) concluded with the poor protection efficacy of a two-dose regimen of the ChAdOx1 nCoV-19 vaccine (AZD1222) against mild-to-moderate COVID-19 due to the B.1.351 variant.

**Table 6:** Influential clinical trials investigating the safety and efficacy of diverse vaccines against SARS-CoV-2 variant.

**Publication** **year**	**Variants**	**Vaccine type**	**Phase**	**No. of** **subjects**	**No. of** patients^*^	**Duration**	**Delivery** **Method**	**Primary clinical measure**	**(Ref.) NCT**
2021	B.1.351 (501Y.V2)	Ad26	III	19,630	86 (SA)	28d	IM	Safety and efficacy of single-dose Ad26.COV2. S Vaccine	(18) 04505722
2021	B.1.351 (501Y.V2)	AZD1222 (ChAdOx1 nCoV-19 vaccine)	III	2,026	42	35d	IM	Efficacy evaluation of two-dose regimen of the ChAdOx1 nCoV-19 vaccine against B.1.351 variants	(19) 04444674
2021	B.1.1.7 (alpha)	AZD1222	II/III	8,534	520	14d	IM	Efficacy evaluation of booster doses of vaccine against B.1.1.7 variants: exploratory analysis	(20) 04400838
2021	B.1.351 (501Y.V2)	BNT162b2 mRNA	III	46,429	9 (SA)	180d	IM	Efficacy evaluation of booster doses of vaccine against B.1.351 variants.	(21) 04368728
2021	B.1.1.7 (alpha)	NVX-CoV2373	III	15,187	8 (VBC)	28d	IM	Efficacy evaluation of two-dose regimen of the ChAdOx1 nCoV-19 vaccine against B.1.1.7 variants	(22) N/A
2021	B.1.351 (501Y.V2)	NVX-CoV2373	II	6,324	38 (SA)	7d	IM	Safety evaluation of ChAdOx1 nCoV-19 vaccine against B.1.351 variants	(23) 04533399

IM, intramuscular injections; BNT162b2, lipid nanoparticle-formulated, nucleoside modified RNA vaccine; Ad26, recombinant adenovirus type-26 vectored SARS-CoV-2 vaccine; AZD1222, novel chimpanzee adenovirus-vectored vaccine, ChAdOx1 nCoV-19; NVX-CoV2373, recombinant severe acute respiratory syndrome coronavirus 2; SA, South Africa; VBC, vaccine breakthrough cases; N/A, not applicable. The symbol “*” presents the number of patients infected with the variants in the whole cohort.

## Discussion

Here, we firstly utilized citation analysis in an attempt to characterize the most cited articles in the SARS-CoV-2 variants field. The number of citations an article has is a valuable measure of its impact on the topics it addresses and this number has become a symbol for evaluating authors and journals (24). Understanding and identification of the features of the 100 most cited articles in SARS-CoV-2 variants domain are of great interest to scientific researchers and the public. First, epidemiologists and infectious disease researchers would find clear study orientations and current challenges that they must face thereby adjusting the therapeutic measures for emerging variants. Furthermore, with the aid of information that our study provides, policymakers also can precisely formulate epidemic prevention and control and variant-associated policies. Third, the public can get research and dissemination of current variants through our studies, which might be helpful for compliance with preventive measures and policies. Finally, our results also can be deemed as a reference for journal editors to evaluate associated scientific works submitted for publication.

During late 2020, the emergence of variants that posed an increased risk to global public health prompted the characterization of specific variants of interest (VOIs) and variants of concern (VOCs). These highlighted the importance of tracking the emergence of mutations in variants genome that impact virulence, enhanced transmissibility, and immune and neutralizing antibody resistance. Therefore, identification and description of genomics information and biological behavior of emerging variants were the working focus in the early works of scientists from diverse research institutions. This trend accordingly promoted the public to think about whether neutralizing antibody responses induced by COVID-19 or current diverse vaccines remain effective. Few phase 1/2 and relatively more phase 3 clinical trials in the 100 most cited articles also explain the increased amount of attention on the vaccine efficacy against variants. Although almost 23% of studies reported the phenomenon of immune escape and poor serum antibodies response, most of the results of RCTs were encouraging. More and large clinical trials are ongoing.

The results of the Spearman correlation and the data distribution of the JIF and CIF ratio in the table demonstrated that many journals with low-impact factors that might be less accessed by the scientific community during a fast-paced pandemic were worthy and very reliable. The top-ranked articles according to the CIF ratio focus more on basic research rather than clinical trials indicating the importance of these articles in guiding clinical decision-making and public health management. Scientific workers and the public should pay more attention to these articles. And based on this, we call for future bibliometric analyses to also adopt this method of data analysis to obtain more valuable conclusions.

In this study, we also summarized the variant types mentioned in the 100 most cited articles and matched them with the topic clusters (Table 6). Mutations in the S protein such as D614G mutation were the dominant variant form in the epidemiology and transmission studies showing that the S protein may be key viral transmission. Identification and description of the spike (S) protein variant D614G are also the hot spots in highly cited articles. The increased infectivity of D614G variants could be associated with the angiotensin-converting enzyme-2 (ACE2) receptor that increased entry efficiency (25). This mutation may be associated with the increase in virulence (or transmissibility) (26) or decrease in effectiveness of public health and social measures. Furthermore, in the vaccine effectiveness and immune escape studies, the B.1.1.7 (501Y.V1, alpha, UK) and B.1.351 (501Y.V2, South Africa) were the most-studied variants. Both of them belong to the previously circulating variants of concern (VOCs) characterized by immune escape and neutralizing antibody resistance (7).

Differentiation and characterization of diverse current variants based on their biological behaviors can aid health workers to periodically adjust the social measures or available diagnostics, vaccines, therapeutics. Variants can be categorized according to the working definition of WHO to their features, mainly including two categories: variants of concern (VOCs) and variants of interest (VOIs). VOCs have been demonstrated to be associated with one or more of the following changes at a degree of global public health significance: increase in transmissibility (virulence), detrimental change in COVID-19 epidemiology, or decrease in the effectiveness of public health and social measures. While VOIs feature the genetic changes that are predicted or known to affect virus characteristics such as transmissibility, disease severity, immune escape, diagnostic or therapeutic escape. The current VOCs and VOIs were summarized in Table 7 based on their Pango lineage, GISAID clade (www.gisaid.org), and Nextstrain clade (http://nextstrain.org/ncov; https://outbreak.info/) information. We believe these pieces of frontier intelligence can provide a lot of information on the current variants and the influence their pose on global health. Systematic approaches to provide a representative indication of the extent of transmission of SARS-CoV-2 variants based on the local context, and to detect unusual epidemiological events can be periodically done.

**Table 7:** Classifications and characterizations of current SARS-CoV-2 variants.

**Variants**	**WHO label**	**Pango lineage**	**GISAID clade**	**Nextstrain clade**	**(Genetic) features**	**Earliest documented samples**
Variants of concern (VOCs)	Alpha	B.1.1.7	GRY	20I (V1)	Characteristic mutations in lineage: ORF1a; ORF1b; S protein; ORF8; N	United Kingdom, Sep-2020
	Beta	B.1.351	GH/501Y.V2	20H (V2)	Characteristic mutations in lineage: ORF1a; ORF1b; S protein; ORF8; N	South Africa, May-2020
	Gamma	P.1	GR/501Y.V3	20J (V3)	Characteristic mutations in lineage: ORF1a; ORF1b; S protein; ORF8; N	Brazil, Nov-2020
	Delta	B.1.617.2	G/478K.V1	21A, 21I, 21J	Characteristic mutations in lineage: ORF1b; S protein; ORF3a (s26L); ORF7a; ORF8; N	India, Oct-2020
	Omicron^*^	B.1.1.529 (total)	GR/484A	21K, 21L, 21M, 22A, 22B, 22C	Additional aino acid changes	Multiple countries, Nov-2021
	Variants under Omicron	BA.4#	GRA22A	22A	BA.2-like constellation in the spike protein + S:del69/70, S:L452R, S:F486V, S:Q493 reversion	South Africa, Jan-2022
		BA.5#	GRA	22B	BA.2-like constellation in the spike protein + S:del69/70, S:L452R, S:F486V, S:Q493 reversion	South Africa, Jan-2022
		BA.2.12.1	GRA	22C	BA.2 + S:L452Q, S:S704F	United States, Dec-2021
		BA.2.9.1 BA.2.13§	GRA	-	BA.2 + S;L452M	Multiple countries, Feb-2022
		BA.2.11^**^	GRA	-	BA.2 + S:L452R	Multiple countries, Mar-2022
Variants of interest (VOIs)	Epsilon	B.1.427 B.1.429	GH/452R.V1	21C	Characteristic mutations in lineage: ORF1a; ORF1b; S protein; N	United States, Mar-2020
	Zeta	P.2	GR/484K.V2	20B/S.484K	Mutations in lineage: ORF1a; ORF1b(P314L); S protein; N	Brazil, Apr-2020
	Eta	B.1.525	G/484K.V3	21D	Mutations in lineage: ORF1a; ORF1b(P314L); S protein; N	Multiple countries, Dec-2020
	Theta	P.3	GR/1092K.V1	21E	Mutations in lineage: ORF1a; ORF1b; S protein; ORF8; N	Philippines, Jan-2021
	Iota	B.1.526	GH/253G.V1	21F	Mutations in lineage: ORF1a; ORF1b; S protein; ORF 3a; ORF8	United States, Nov-2020
	Kappa	B.1.617.1	G/452R.V3	21B	Mutations: ORF1a; ORF1b; S protein; ORF 3a; ORF8	India, Oct-2020
	Lambda	C.37	GR/452Q.V1	21G	Mutations in lineage: ORF1a; ORF1b; S protein; ORF8; N	Peru, Dec-2020
	Mu	B.1.621	GH	21H	Mutations in lineage: ORF1a; ORF1b; S protein; ORF 3a; ORF8	Colombia, Jan-2021

* Includes BA.1, BA.2, BA.3, BA.4, BA.5 and descendent lineages; ^#^these lineages have identical constellation of mutations in the spike and the following differences outside the spike: BA.4: ORF7b:L11F, N:P151S; BA.5: M:D3N. Both have reversions at nsp4: L438 and ORF6:D61;^§^these lineages have identical constellation of mutations in the spike and the following differences outside the spike: BA.2.9.1: ORF3a:H78Y, N: P67S, N: S412I;

** additional mutation outside the spike protein: ORF1a:S2519P.

## Limitations

This study has limitations. Relying on only citation analysis maybe not be a perfect approach to evaluating the quality of articles on a specific field. Factors that influence citations an article possess involve not only publication-quality but also other factors that are not accounted for in our study such as self-citation and obliteration by incorporation (27). Another limitation is that the searching terms we used are highly dependent on the NLM's Medical Subject Headings (MeSH), which may lead to incomplete retrieval of publications in SARS-CoV-2 variant domain. Third, the presence of the phenomenon of anonymity that cannot be settled currently in the co-cited analysis of authors maybe make the results controversial. Finally, the citation database bias. We used only the Web of Science database to calculate the number of citations of articles in the current study that might differ from other databases such as PubMed, Scopus, and Embase databases.

## Conclusion

SARS-CoV-2 is evolving. Genomics information, spike protein structure confirmation and neutralization studies on the evaluation of antibody resistance were highly represented in the 100 most cited articles in SARS-CoV-2 variants literature. Mutations in the S protein such as D614G mutation and the B.1.1.7 (alpha, UK) and B.1.351 (South Africa) were the dominant variants in the 100 most cited articles. Generally speaking, the responses of vaccine-induced antibody binding and neutralization against the SARS-CoV-2 were weakened due to the presence of variants, but the results of clinical trials were encouraging. Differentiation and characterization of diverse current variants based on their biological behaviors can encourage authorities to strengthen surveillance and sequencing capacities and apply systematic approaches to control them. Given the continuous evolution of the SARS-CoV-2 and the constant development in our understanding of the impact of variants, current working strategies and measures may be periodically adjusted.

## Data availability statement

The original contributions presented in the study are included in the article/Supplementary material, further inquiries can be directed to the corresponding author.

## Author contributions

RM contributed to the study conception and design. YZ and MF performed material preparation, data collection, and analysis. YZ finished the first draft of the manuscript. YH and FL commented on previous versions of the manuscript. All authors read and approved the final manuscript.

## Conflict of interest

The authors declare that the research was conducted in the absence of any commercial or financial relationships that could be construed as a potential conflict of interest.

## Publisher's note

All claims expressed in this article are solely those of the authors and do not necessarily represent those of their affiliated organizations, or those of the publisher, the editors and the reviewers. Any product that may be evaluated in this article, or claim that may be made by its manufacturer, is not guaranteed or endorsed by the publisher.
