# Supplementary material for: A bibliometric analysis of the 100 most cited articles describing SARS-CoV-2 variants
Source: Front Public Health. 2022 Aug 26;10:966847. doi: 10.3389/fpubh.2022.966847 (PMC9458909; doi:10.3389/fpubh.2022.966847)
Supplement: Supplementary file 1 [file Data_Sheet_1.docx]

**Supplement Materials**

**Number of Supplements: 2**

**Supplemental Table 1** The 100 most-cited SARS-CoV-2 variant articles ranked in order of the number of citations received.

**Table 1 The 100 most-cited SARS-CoV-2 variants articles ranked in order of the number of citations received.**

| **Rank** | **Article** | **Type of Article** | **Date** | **No. of citations** | **Citations over year** | | | **Author (first)** | **Journal** |
| --- | --- | --- | --- | --- | --- | --- | --- | --- | --- |
|  |  |  |  |  | **2020** | **2021** | **2022** |  |  |
| 1 | Tracking Changes in SARS-CoV-2 Spike: Evidence that D614G Increases Infectivity of the COVID-19 Virus | basic research | 2020 | 1720 | 239 | 1199 | 282 | Korber, B | Cell |
| 2 | Antibody resistance of SARS-CoV-2 variants B.1.351 and B.1.1.7 | basic research | 2021 | 894 | 0 | 630 | 264 | Wang, PF | Nature |
| 3 | Effectiveness of Covid-19 Vaccines against the B.1.617.2 (Delta) Variant | Case-control design | 2021 | 870 | 0 | 429 | 442 | Bernal, JL | NEJM |
| 4 | Inborn errors of type I IFN immunity in patients with life-threatening COVID-19 | basic research | 2020 | 786 | 58 | 596 | 132 | Zhang, Q | Science |
| 5 | Estimated transmissibility and impact of SARS-CoV-2 lineage B.1.1.7 in England | basic research | 2021 | 781 | 1 | 534 | 246 | Davies, NG | Science |
| 6 | Neutralizing antibody levels are highly predictive of immune protection from symptomatic SARS-CoV-2 infection | basic research | 2021 | 739 | 0 | 361 | 378 | Khoury, DS | Nature Medicine |
| 7 | A Multibasic Cleavage Site in the Spike Protein of SARS-CoV-2 Is Essential for Infection of Human Lung Cells | basic research | 2020 | 720 | 182 | 416 | 122 | Hoffmann, M | Molecular Cell |
| 8 | The Impact of Mutations in SARS-CoV-2 Spike on Viral Infectivity and Antigenicity | basic research | 2020 | 638 | 44 | 467 | 128 | Li, QQ | Cell |
| 9 | Detection of a SARS-CoV-2 variant of concern in South Africa | basic research | 2021 | 624 | 0 | 446 | 178 | Tegally, H | Nature |
| 10 | Safety and Efficacy of Single-Dose Ad26.COV2.S Vaccine against Covid-19 | Clinical trial | 2021 | 603 | 2 | 326 | 275 | Sadoff, J | NEJM |
| 11 | Spike mutation D614G alters SARS-CoV-2 fitness | basic research | 2021 | 601 | 13 | 460 | 128 | Plante, JA | Nature |
| 12 | COVID-19 vaccine BNT162b1 elicits human antibody and T(H)1 T cell responses | basic research | 2020 | 556 | 16 | 433 | 107 | Sahin, U | Nature |

**Table** **1 The 100 most-cited SARS-CoV-2 variants articles ranked in order of the number of citations received. *(Continued)***

| **Rank** | **Article** | **Type of Article** | **Date** | **No. of citations** | **Citations over year** | | | **Author (first)** | **Journal** |
| --- | --- | --- | --- | --- | --- | --- | --- | --- | --- |
|  |  |  |  |  | **2020** | **2021** | **2022** |  |  |
| 13 | SARS-CoV-2 501Y.V2 escapes neutralization by South African COVID-19 donor plasma | basic research | 2021 | 542 | 0 | 423 | 119 | Wibmer, CK | Nature Medicine |
| 14 | Antibody cocktail to SARS-CoV-2 spike protein prevents rapid mutational escape seen with individual antibodies | basic research | 2020 | 526 | 63 | 375 | 88 | Baum, A | Science |
| 15 | Phylogenetic network analysis of SARS-CoV-2 genomes | basic research | 2020 | 484 | 221 | 231 | 32 | Forster, P | PNASUSA |
| 16 | Efficacy of the ChAdOx1 nCoV-19 Covid-19 Vaccine against the B.1.351 Variant | Clinical trial | 2021 | 480 | 1 | 345 | 134 | Madhi, SA | NEJM |
| 17 | Emerging SARS-CoV-2 mutation hot spots include a novel RNA-dependent-RNA polymerase variant | basic research | 2020 | 433 | 132 | 253 | 48 | Pachetti, M | Journal of Translational Medicine |
| 18 | Genomics and epidemiology of the P.1 SARS-CoV-2 lineage in Manaus, Brazil | basic research | 2021 | 410 | 1 | 284 | 125 | Faria, NR | Science |
| 19 | Evidence of escape of SARS-CoV-2 variant B.1.351 from natural and vaccine-induced sera | basic research | 2021 | 381 | 0 | 249 | 132 | Zhou, D | Cell |
| 20 | Escape from neutralizing antibodies by SARS-CoV-2 spike protein variants | basic research | 2020 | 350 | 2 | 240 | 108 | Weisblum, Y | Elife |
| 21 | SARS-CoV-2 variants B.1.351 and P.1 escape from neutralizing antibodies | basic research | 2021 | 338 | 0 | 226 | 112 | Hoffmann, M | Cell |
| 22 | Covid-19 Breakthrough Infections in Vaccinated Health Care Workers | basic research | 2021 | 330 | 0 | 120 | 210 | Bergwerk, M | NEJM |
| 23 | Resistance of SARS-CoV-2 variants to neutralization by monoclonal and serum-derived polyclonal antibodies | basic research | 2021 | 330 | 0 | 229 | 101 | Chen, RE | Nature Medicine |

**Table 1 The 100 most-cited SARS-CoV-2 variants articles ranked in order of the number of citations received. *(Continued)***

| **Rank** | **Article** | **Type of Article** | **Date** | **No. of citations** | **Citations over year** | | | **Author (first)** | **Journal** |
| --- | --- | --- | --- | --- | --- | --- | --- | --- | --- |
|  |  |  |  |  | **2020** | **2021** | **2022** |  |  |
| 24 | Presence of Genetic Variants Among Young Men With Severe COVID-19 | basic research | 2020 | 313 | 43 | 210 | 60 | Van DM | JAMA |
| 25 | Sensitivity of SARS-CoV-2 B.1.1.7 to mRNA vaccine-elicited antibodies | basic research | 2021 | 292 | 0 | 206 | 86 | Collier, DA | Nature |
| 26 | Genomic Diversity of Severe Acute Respiratory Syndrome-Coronavirus 2 in Patients With Coronavirus Disease 2019 | basic research | 2020 | 272 | 88 | 164 | 20 | Shen, ZJ | Clinical Infectious Disease |
| 27 | Neutralization of SARS-CoV-2 spike 69/70 deletion, E484K and N501Y variants by BNT162b2 vaccine-elicited sera | basic research | 2021 | 264 | 1 | 180 | 83 | Xie, XP | Nature Medicine |
| 28 | Evaluating the Effects of SARS-CoV-2 Spike Mutation D614G on Transmissibility and Pathogenicity | basic research | 2021 | 263 | 0 | 182 | 81 | Volz, E | Cell |
| 29 | Neutralization of SARS-CoV-2 lineage B.1.1.7 pseudovirus by BNT162b2 vaccine-elicited human sera | basic research | 2021 | 257 | 0 | 208 | 49 | Muik, A | Science |
| 30 | Vaccine Breakthrough Infections with SARS-CoV-2 Variants | basic research | 2021 | 250 | 1 | 144 | 105 | Hacisuleyman | NEJM |
| 31 | Case Study: Prolonged Infectious SARS-CoV-2 Shedding from an Asymptomatic Immunocompromised Individual with Cancer | basic research | 2020 | 245 | 1 | 201 | 43 | Avanzato, VA. | Cell |
| 32 | SARS-CoV-2 D614G variant exhibits efficient replication ex vivo and transmission in vivo | basic research | 2020 | 242 | 0 | 165 | 77 | Hou, YJ. | Science |
| 33 | Structural and Functional Analysis of the D614G SARS-CoV-2 Spike Protein Variant | basic research | 2020 | 239 | 10 | 146 | 83 | Yurkovetskiy | Cell |
| 34 | Variant analysis of SARS-CoV-2 genomes | basic research | 2020 | 229 | 22 | 161 | 46 | Koyama, T | BOWHO |
| 35 | Circulating SARS-CoV-2 spike N439K variants maintain fitness while evading antibody-mediated immunity | basic research | 2021 | 226 | 0 | 172 | 54 | Thomson, EC | Cell |

**Table 1 The 100 most-cited SARS-CoV-2 variants articles ranked in order of the number of citations received. *(Continued)***

| **Rank** | **Article** | **Type of Article** | **Date** | **No. of citations** | **Citations over year** | | | **Author (first)** | **Journal** |
| --- | --- | --- | --- | --- | --- | --- | --- | --- | --- |
|  |  |  |  |  | **2020** | **2021** | **2022** |  |  |
| 36 | Efficacy of ChAdOx1 nCoV-19 (AZD1222) vaccine against SARS-CoV-2 variant of concern 202012/01 (B.1.1.7): an exploratory analysis of a randomised controlled trial | Clinical trial | 2021 | 217 | 0 | 136 | 81 | Emary, KRW | Lancet |
| 37 | Identification of SARS-CoV-2 spike mutations that attenuate monoclonal and serum antibody neutralization | basic research | 2020 | 216 | 0 | 146 | 70 | Liu, ZM | Cell host & Microbe |
| 38 | SARS-CoV-2 spike-protein D614G mutation increases virion spike density and infectivity | basic research | 2021 | 214 | 0 | 166 | 48 | Zhang, LZ | Nature Communications |
| 39 | Escape of SARS-CoV-2 501Y.V2 from neutralization by convalescent plasma | basic research | 2021 | 213 | 0 | 153 | 60 | Cele, S | Nature |
| 40 | Sensitivity of infectious SARS-CoV-2 B.1.1.7 and B.1.351 variants to neutralizing antibodies | basic research | 2021 | 207 | 0 | 113 | 94 | Planas, D | Nature Medicine |
| 41 | Antibody evasion by the P.1 strain of SARS-CoV-2 | basic research | 2021 | 203 | 0 | 135 | 68 | Dejnirattisai | Cell |
| 42 | SARS-CoV-2 evolution during treatment of chronic infection | basic research | 2021 | 201 | 0 | 132 | 69 | Kemp, SA. | Nature |
| 43 | Reduced neutralization of SARS-CoV-2 B.1.1.7 variant by convalescent and vaccine sera | basic research | 2020 | 198 | 21 | 141 | 36 | Supasa, P | Cell |
| 44 | Engineering human ACE2 to optimize binding to the spike protein of SARS coronavirus 2 | basic research | 2020 | 192 | 7 | 136 | 49 | Chan, KK | Science |
| 45 | Mutations Strengthened SARS-CoV-2 Infectivity | basic research | 2021 | 217 | 0 | 136 | 81 | Chen, JH | Journal of Molecular Biology |
| 46 | Effects of a major deletion in the SARS-CoV-2 genome on the severity of infection and the inflammatory response: an observational cohort study | observational cohort study | 2020 | 191 | 20 | 147 | 24 | Young, BE | Lancet |

**Table 1 The 100 most-cited SARS-CoV-2 variants articles ranked in order of the number of citations received. *(Continued)***

| **Rank** | **Article** | **Type of Article** | **Date** | **No. of citations** | **Citations over year** | | | **Author (first)** | **Journal** |
| --- | --- | --- | --- | --- | --- | --- | --- | --- | --- |
|  |  |  |  |  | **2020** | **2021** | **2022** |  |  |
| 47 | SARS-CoV-2 B.1.617.2 Delta variant replication and immune evasion | basic research | 2021 | 187 | 1 | 49 | 137 | Mlcochova, P | Nature |
| 48 | Recurrent deletions in the SARS-CoV-2 spike glycoprotein drive antibody escape | basic research | 2021 | 182 | 1 | 126 | 55 | McCarthy, KR | Science |
| 49 | Structural variations in human ACE2 may influence its binding with SARS-CoV-2 spike protein | basic research | 2020 | 182 | 79 | 88 | 15 | Hussain, M | Journal of Medical Virology |
| 50 | Reduced neutralization of SARS-CoV-2 B.1.617 by vaccine and convalescent serum | basic research | 2021 | 180 | 0 | 83 | 97 | Liu, C | Cell |
| 51 | Safety and Efficacy of the BNT162b2 mRNA Covid-19 Vaccine through 6 Months | Clinical trial | 2021 | 177 | 0 | 43 | 134 | Thomas, SJ | NEJM |
| 52 | SARS-CoV-2 ORF3b Is a Potent Interferon Antagonist Whose Activity Is Increased by a Naturally Occurring Elongation Variant | basic research | 2020 | 173 | 26 | 117 | 30 | Konno, Y | Cell Reports |
| 53 | Safety and Efficacy of NVX-CoV2373 Covid-19 Vaccine | Clinical trial | 2021 | 171 | 0 | 84 | 87 | Heath, P T | NEJM |
| 54 | Transmission, infectivity, and neutralization of a spike L452R SARS-CoV-2 variant | basic research | 2021 | 170 | 0 | 107 | 63 | Deng, XD | Cell |
| 55 | Correlates of protection against symptomatic and asymptomatic SARS-CoV-2 infection | basic research | 2021 | 162 | 0 | 39 | 123 | Feng, S | Nature Medicine |
| 56 | Naturally enhanced neutralizing breadth against SARS-CoV-2 one year after infection | basic research | 2021 | 161 | 0 | 80 | 81 | Wang, ZJ | Nature |
| 57 | Efficacy of NVX-CoV2373 Covid-19 Vaccine against the B.1.351 Variant | Clinical trial | 2021 | 161 | 0 | 111 | 50 | Shinde, V | NEJM |

**Table 1 The 100 most-cited SARS-CoV-2 variants articles ranked in order of the number of citations received. *(Continued)***

| **Rank** | **Article** | **Type of Article** | **Date** | **No. of citations** | **Citations over year** | | | **Author (first)** | **Journal** |
| --- | --- | --- | --- | --- | --- | --- | --- | --- | --- |
|  |  |  |  |  | **2020** | **2021** | **2022** |  |  |
| 58 | SARS-CoV-2 genomic variations associated with mortality rate of COVID-19 | basic research | 2020 | 159 | 15 | 120 | 24 | Toyoshima, YJ | Journal of Human Genetics |
| 59 | ACE2 and TMPRSS2 variants and expression as candidates to sex and country differences in COVID-19 severity in Italy | observational cohort study | 2020 | 154 | 41 | 89 | 24 | Asselta, R | Aging-us |
| 60 | mRNA vaccination boosts cross-variant neutralizing antibodies elicited by SARS-CoV-2 infection | basic research | 2021 | 152 | 0 | 88 | 64 | Stamatatos, L | Science |
| 61 | Sixteen novel lineages of SARS-CoV-2 in South Africa | basic research | 2021 | 150 | 0 | 114 | 36 | Tegally, H | Nature Medicine |
| 62 | Virus-Receptor Interactions of Glycosylated SARS-CoV-2 Spike and Human ACE2 Receptor | basic research | 2020 | 145 | 15 | 102 | 28 | Zhao, P | Cell host & Microbe |
| 63 | SARS-CoV-2 D614G spike mutation increases entry efficiency with enhanced ACE2-binding affinity | basic research | 2021 | 143 | 0 | 95 | 48 | Ozono, SY | Nature Communications |
| 64 | Rapid reconstruction of SARS-CoV-2 using a synthetic genomics platform | basic research | 2020 | 143 | 33 | 83 | 27 | Tran TNT | Nature |
| 65 | Characterisation of the transcriptome and proteome of SARS-CoV-2 reveals a cell passage induced in-frame deletion of the furin-like cleavage site from the spike glycoprotein | basic research | 2020 | 141 | 26 | 94 | 21 | Davidson, AD. | Genome Medicine |
| 66 | SARS-CoV-2 variants of concern partially escape humoral but not T- cell responses in COVID-19 convalescent donors and vaccinees | basic research | 2021 | 138 | 0 | 66 | 72 | Geers, D | Science Immunology |
| 67 | SARS-CoV-2 501Y.V2 variants lack higher infectivity but do have immune escape | basic research | 2021 | 135 | 0 | 75 | 60 | Li, QQ | Cell |

**Table 1 The 100 most-cited SARS-CoV-2 variants articles ranked in order of the number of citations received. *(Continued)***

| **Rank** | **Article** | **Type of Article** | **Date** | **No. of citations** | **Citations over year** | | | **Author (first)** | **Journal** |
| --- | --- | --- | --- | --- | --- | --- | --- | --- | --- |
|  |  |  |  |  | **2020** | **2021** | **2022** |  |  |
| 68 | Attenuated SARS-CoV-2 variants with deletions at the S1/S2 junction | basic research | 2020 | 135 | 36 | 80 | 19 | Lau, SY | Emerging Microbes & Infections |
| 69 | Genomic characteristics and clinical effect of the emergent SARS-CoV-2 B.1.1.7 lineage in London, UK: a whole-genome sequencing and hospital-based cohort study | hospital-based cohort study | 2021 | 133 | 0 | 94 | 39 | Frampton, D | lancet infectious diseases |
| 70 | Waning Immunity after the BNT162b2 Vaccine in Israel | basic research | 2021 | 127 | 0 | 12 | 116 | Goldberg, Y | NEJM |
| 71 | Waning of BNT162b2 Vaccine Protection against SARS-CoV-2 Infection in Qatar | basic research | 2021 | 123 | 0 | 33 | 90 | Chemaitelly, H | NEJM |
| 72 | SARS-CoV-2 spike D614G change enhances replication and transmission | basic research | 2021 | 115 | 0 | 67 | 48 | Zhou, B | Nature |
| 73 | Discovery and Genomic Characterization of a 382-Nucleotide Deletion in ORF7b and ORF8 during the Early Evolution of SARS-CoV-2 | basic research | 2020 | 115 | 16 | 87 | 12 | Su, YCF | Mbio |
| 74 | Emergence of Drift Variants That May Affect COVID-19 Vaccine Development and Antibody Treatment | basic research | 2020 | 115 | 27 | 74 | 14 | Koyama, T | Pathogens |
| 75 | ACE2gene variants may underlie interindividual variability and susceptibility to COVID-19 in the Italian population | basic research | 2020 | 108 | 23 | 69 | 16 | Benetti, E | European Journal of Human Genetics |
| 76 | Immune responses against SARS-CoV-2 variants after heterologous and homologous ChAdOx1 nCoV-19/BNT162b2 vaccination | basic research | 2021 | 106 | 0 | 54 | 52 | Barros-MJ | Nature Medicine |

**Table 1 The 100 most-cited SARS-CoV-2 variants articles ranked in order of the number of citations received. *(Continued)***

| **Rank** | **Article** | **Type of Article** | **Date** | **No. of citations** | **Citations over year** | | | **Author (first)** | **Journal** |
| --- | --- | --- | --- | --- | --- | --- | --- | --- | --- |
|  |  |  |  |  | **2020** | **2021** | **2022** |  |  |
| 77 | Structural impact on SARS-CoV-2 spike protein by D614G substitution | basic research | 2021 | 106 | 0 | 54 | 52 | Zhang, J | Science |
| 78 | Increased resistance of SARS-CoV-2 variant P.1 to antibody neutralization | basic research | 2021 | 105 | 0 | 58 | 47 | Wang, PF | Cell host & Microbe |
| 79 | Age-related immune response heterogeneity to SARS-CoV-2 vaccine BNT162b2 | basic research | 2021 | 104 | 0 | 44 | 60 | Collier, DA | Nature |
| 80 | Recurrent emergence of SARS-CoV-2 spike deletion H69/V70 and its role in the Alpha variant B.1.1.7 | basic research | 2021 | 102 | 0 | 56 | 46 | Meng, B | Cell Reports |
| 81 | A SARS-CoV-2 vaccine candidate would likely match all currently circulating variants | basic research | 2020 | 101 | 4 | 79 | 18 | Dearlove, B | PNASUSA |
| 82 | SARS-CoV-2 Spike Mutations, L452R, T478K, E484Q and P681R, in the Second Wave of COVID-19 in Maharashtra, India | basic research | 2021 | 100 | 0 | 48 | 52 | Cherian, S | Microorganisms |
| 83 | SARS-CoV-2 variant B.1.617 is resistant to bamlanivimab and evades antibodies induced by infection and vaccination | basic research | 2021 | 99 | 0 | 60 | 39 | Hoffmann, M | Cell Reports |
| 84 | Higher infectivity of the SARS-CoV-2 new variants is associated with K417N/T, E484K, and N501Y mutants: An insight from structural data | basic research | 2021 | 99 | 0 | 67 | 32 | Khan, A | Journal of Cellular Physiology |
| 85 | Durability of mRNA-1273 vaccine-induced antibodies against SARS-CoV-2 variants | basic research | 2021 | 98 | 0 | 29 | 69 | Pegu, A | Science |
| 86 | Prior SARS-CoV-2 infection rescues B and T cell responses to variants after first vaccine dose | basic research | 2021 | 98 | 0 | 59 | 39 | Reynolds, CJ | Science |

**Table 1 The 100 most-cited SARS-CoV-2 variants articles ranked in order of the number of citations received. *(Continued)***

| **Rank** | **Article** | **Type of Article** | **Date** | **No. of citations** | **Citations over year** | | | **Author (first)** | **Journal** |
| --- | --- | --- | --- | --- | --- | --- | --- | --- | --- |
|  |  |  |  |  | **2020** | **2021** | **2022** |  |  |
| 87 | Novel SARS-CoV-2 variants: the pandemics within the pandemic | basic research | 2021 | 95 | 0 | 54 | 41 | Boehm, E | Clinical Microbiology and Infection |
| 88 | BNT162b2-elicited neutralization of B.1.617 and other SARS-CoV-2 variants | basic research | 2021 | 93 | 0 | 62 | 31 | Liu, JY | Nature |
| 89 | Effect of Delta variant on viral burden and vaccine effectiveness against new SARS-CoV-2 infections in the UK | basic research | 2021 | 91 | 0 | 15 | 76 | Pouwels, KB | Nature Medicine |
| 90 | D614G Mutation Alters SARS-CoV-2 Spike Conformation and Enhances Protease Cleavage at the S1/S2 Junction | basic research | 2021 | 91 | 0 | 68 | 23 | Gobeil, SMC | Cell Reports |
| 91 | Community transmission and viral load kinetics of the SARS-CoV-2 delta (B.1.617.2) variant in vaccinated and unvaccinated individuals in the UK: a prospective, longitudinal, cohort study | longitudinal, cohort study | 2022 | 90 | 0 | 9 | 81 | Singanayagam, A | Lancet Infectious Diseases |
| 92 | Omicron extensively but incompletely escapes Pfizer BNT162b2 neutralization | basic research | 2022 | 85 | 0 | 3 | 82 | Cele, S | Nature |
| 93 | Effect of natural mutations of SARS-CoV-2 on spike structure, conformation, and antigenicity | basic research | 2021 | 85 | 0 | 47 | 38 | Gobeil, SMC | Science |
| 94 | COVID-19 in Amazonas, Brazil, was driven by the persistence of endemic lineages and P.1 emergence | basic research | 2021 | 85 | 0 | 50 | 35 | Naveca, FG | Nature Medicine |
| 95 | SARS-CoV-2 escape from a highly neutralizing COVID-19 convalescent plasma | basic research | 2021 | 84 | 0 | 42 | 42 | Andreano, E | PNASUSA |

**Table 1 The 100 most-cited SARS-CoV-2 variants articles ranked in order of the number of citations received. *(Continued)***

| **Rank** | **Article** | **Type of Article** | **Date** | **No. of citations** | **Citations over year** | | | **Author (first)** | **Journal** |
| --- | --- | --- | --- | --- | --- | --- | --- | --- | --- |
|  |  |  |  |  | **2020** | **2021** | **2022** |  |  |
| 96 | SARS-CoV-2 spike L452R variant evades cellular immunity and increases infectivity | basic research | 2021 | 84 | 0 | 38 | 46 | Motozono, C | Cell host & Microbe |
| 97 | SARS-CoV-2 Transmission between Mink (Neovison vison) and Humans, Denmark | basic research | 2021 | 83 | 1 | 68 | 14 | Hammer, AS | Emerging Infectious Diseases |
| 98 | Infection- and vaccine-induced antibody binding and neutralization of the B.1.351 SARS-CoV-2 variant | basic research | 2021 | 77 | 0 | 44 | 33 | Edara, VV | Cell host & Microbe |
| 99 | SARS-CoV-2 within-host diversity and transmission | basic research | 2021 | 76 | 0 | 49 | 27 | Lythgoe, KA | Science |
| 100 | mRNA vaccines induce durable immune memory to SARS-CoV-2 and variants of concern | basic research | 2021 | 75 | 0 | 9 | 66 | Goel, RR | Science |

NEJM, New England Journal of Medicine; PNASUSA, Proceedings of the National Academy of Sciences of the United States of America; BOWHO, Bulletin of the World Health Organization.

**Table 2 The 20 most-cited SARS-CoV-2 variants articles ranked in order of the CIF (number of citations/journal impact factor) ratio received.**

| **Rank**  **(rank*)** | **Article** | **Type of Article** | **Date** | **No. of citations** | **Citations over year** | | | **Author (first)** | **Journal**  **(IF 2020)** |
| --- | --- | --- | --- | --- | --- | --- | --- | --- | --- |
|  |  |  |  |  | **2020** | **2021** | **2022** |  |  |
| 1  (17) | Emerging SARS-CoV-2 mutation hot spots include a novel RNA-dependent-RNA polymerase variant | basic research | 2020 | 433 | 132 | 253 | 48 | Pachetti, M | Journal of Translational Medicine (5.531) |
| 2  (49) | Structural variations in human ACE2 may influence its binding with SARS-CoV-2 spike protein | basic research | 2020 | 182 | 79 | 88 | 15 | Hussain, M | Journal of Medical Virology (2.327) |
| 3  (58) | SARS-CoV-2 genomic variations associated with mortality rate of COVID-19 | basic research | 2020 | 159 | 15 | 120 | 24 | Toyoshima, YJ | Journal of Human Genetics (3.172) |
| 4 (15) | Phylogenetic network analysis of SARS-CoV-2 genomes | basic research | 2020 | 484 | 221 | 231 | 32 | Forster, P | PNASUSA (11.205) |
| 5  (20) | Escape from neutralizing antibodies by SARS-CoV-2 spike protein variants | basic research | 2020 | 350 | 2 | 240 | 108 | Weisblum, Y | Elife  (8.146) |
| 6  (1) | Tracking Changes in SARS-CoV-2 Spike: Evidence that D614G Increases Infectivity of the COVID-19 Virus | basic research | 2020 | 1720 | 239 | 1199 | 282 | Korber, B | Cell  (41.584) |
| 7  (7) | A Multibasic Cleavage Site in the Spike Protein of SARS-CoV-2 Is Essential for Infection of Human Lung Cells | basic research | 2020 | 720 | 182 | 416 | 122 | Hoffmann, M | Molecular Cell  (17.97) |
| 8  (45) | Mutations Strengthened SARS-CoV-2 Infectivity | basic research | 2021 | 217 | 0 | 136 | 81 | Chen, JH | Journal of Molecular Biology (5.469) |
| 9  (74) | Emergence of Drift Variants That May Affect COVID-19 Vaccine Development and Antibody Treatment | basic research | 2020 | 115 | 27 | 74 | 14 | Koyama, T | Pathogens  (3.492) |

**Table 2 The 20 most-cited SARS-CoV-2 variants articles ranked in order of the CIF ratio received *(continued).***

| **Rank** | **Article** | **Type of Article** | **Date** | **No. of citations** | **Citations over year** | | | **Author (first)** | **Journal** |
| --- | --- | --- | --- | --- | --- | --- | --- | --- | --- |
|  |  |  |  |  | **2020** | **2021** | **2022** |  |  |
| 10  (26) | Genomic Diversity of Severe Acute Respiratory Syndrome-Coronavirus 2 in Patients With Coronavirus Disease 2019 | basic research | 2020 | 272 | 88 | 164 | 20 | Shen, ZJ | Clinical Infectious Disease (9.079) |
| 11  (59) | ACE2 and TMPRSS2 variants and expression as candidates to sex and country differences in COVID-19 severity in Italy | observational cohort study | 2020 | 154 | 41 | 89 | 24 | Asselta, R | Aging-us  (5.682) |
| 12  (75) | ACE2gene variants may underlie interindividual variability and susceptibility to COVID-19 in the Italian population | basic research | 2020 | 108 | 23 | 69 | 16 | Benetti, E | European Journal of Human Genetics  (4.246) |
| 13(34) | Variant analysis of SARS-CoV-2 genomes | basic research | 2020 | 229 | 22 | 161 | 46 | Koyama, T | BOWHO  (9.408) |
| 14  (82) | SARS-CoV-2 Spike Mutations, L452R, T478K, E484Q and P681R, in the Second Wave of COVID-19 in Maharashtra, India | basic research | 2021 | 100 | 0 | 48 | 52 | Cherian, S | Microorganisms  (4.128) |
| 15  (68) | Attenuated SARS-CoV-2 variants with deletions at the S1/S2 junction | basic research | 2020 | 135 | 36 | 80 | 19 | Lau, SY | Emerging Microbes & Infections  (7.163) |

**Table 2 The 20 most-cited SARS-CoV-2 variants articles ranked in order of the CIF ratio received *(continued).***

| **Rank** | **Article** | **Type of Article** | **Date** | **No. of citations** | **Citations over year** | | | **Author (first)** | **Journal** |
| --- | --- | --- | --- | --- | --- | --- | --- | --- | --- |
|  |  |  |  |  | **2020** | **2021** | **2022** |  |  |
| 16  (52) | SARS-CoV-2 ORF3b Is a Potent Interferon Antagonist Whose Activity Is Increased by a Naturally Occurring Elongation Variant | basic research | 2020 | 173 | 26 | 117 | 30 | Konno, Y | Cell Reports  (9.423) |
| 17  (2) | Antibody resistance of SARS-CoV-2 variants B.1.351 and B.1.1.7 | basic research | 2021 | 894 | 0 | 630 | 264 | Wang, PF | Nature  (49.962) |
| 18  (4) | Inborn errors of type I IFN immunity in patients with life-threatening COVID-19 | basic research | 2020 | 786 | 58 | 596 | 132 | Zhang, Q | Science  (47.728) |
| 19  (5) | Estimated transmissibility and impact of SARS-CoV-2 lineage B.1.1.7 in England | basic research | 2021 | 781 | 1 | 534 | 246 | Davies, NG | Science  (47.728) |
| 20  (84) | Higher infectivity of the SARS-CoV-2 new variants is associated with K417N/T, E484K, and N501Y mutants: An insight from structural data | basic research | 2021 | 99 | 0 | 67 | 32 | Khan, A | Journal of Cellular Physiology  (6.384) |

BOWHO, Bulletin of the World Health Organization; *: the rank of articles ranked in order of the number of citations in order of the number of citations in the supplementary table 1.
